# The Role of High-Flow Nasal Cannula Oxygen Therapy in Exercise Testing and Pulmonary Rehabilitation: A Review of the Current Literature

**DOI:** 10.3390/jcm13010232

**Published:** 2023-12-30

**Authors:** Claudio Candia, Carmen Lombardi, Claudia Merola, Pasquale Ambrosino, Silvestro Ennio D’Anna, Aldo Vicario, Stefania De Marco, Antonio Molino, Mauro Maniscalco

**Affiliations:** 1 Department of Clinical Medicine and Surgery, University of Naples “Federico II”, 80131 Naples, Italy; claudio.candia@unina.it (C.C.); aldo.vicario@unina.it (A.V.); antonio.molino@unina.it (A.M.); 2 Istituti Clinici Scientifici Maugeri IRCCS, Pulmonary Rehabilitation Unit of Telese Terme Institute, 82037 Telese Terme, Italy; carmen.lombardi@icsmaugeri.it (C.L.); claudia.merola@icsmaugeri.it (C.M.); silvestro.danna@icsmaugeri.it (S.E.D.); 3 Istituti Clinici Scientifici Maugeri IRCCS, Directorate of Telese Terme Institute, 82037 Telese Terme, Italy; pasquale.ambrosino@icsmaugeri.it

**Keywords:** high-flow nasal cannula, pulmonary rehabilitation, exercise training, COPD, ILDs

## Abstract

High-flow nasal cannula (HFNC) has recently emerged as a crucial therapeutic strategy for hypoxemic patients both in acute and chronic settings. Indeed, HFNC therapy is able to deliver higher fractions of inspired oxygen (FiO_2_) with a heated and humidified gas flow ranging from 20 up to 60 L per minute, in a more comfortable way for the patient in comparison with Conventional Oxygen Therapy (COT). In fact, the flow keeps the epithelium of the airways adequately moisturized, thus positively affecting the mucus clearance. Finally, the flow is able to wash out the carbon dioxide in the dead space of the airways; this is also enhanced by a modest positive end-expiratory pressure (PEEP) effect. Recent evidence has shown applications of HFNC in exercise training and chronic settings with promising results. In this narrative review, we explored how HFNC might contribute to enhancing outcomes of exercise training and pulmonary rehabilitation among patients dealing with chronic obstructive pulmonary disease, interstitial lung diseases, and lung cancer.

## 1. Introduction

High-flow nasal cannula (HFNC) has recently emerged as a crucial therapeutic support strategy for hypoxemic patients [1]. The effects of HFNC on the airways and lung mechanics have been extensively described [2,3]. In brief, through the generation of a humidified and heated flow of medical gas, whose Fraction of Inhaled Oxygen (FiO_2_) can be easily regulated and monitored, the device is able to:

- humidify the epithelium of the airways, thus improving ciliary motion and mucus clearance [4];
- wash out the dead space, removing excess carbon dioxide (CO_2_), thus improving hypercapnia [5];
- reduce the work of breathing [6];
- generate a small end-expiratory positive pressure (PEEP), which helps wash out CO_2_ and prevents the airways from collapsing during the expiratory phase [7].

In addition, in comparison with Non-Invasive Ventilation (NIV) and Conventional Oxygen Therapy (COT), HFNC is often perceived as more comfortable by patients [4]. Therefore, HFNC has been increasingly used in clinical practice for several different respiratory conditions, from bronchiolitis in children [8] to acute respiratory failure (ARF) in adults [9,10], with a crucial role during the COVID-19 pandemic in patients with different severities of hypoxemia [11,12,13,14]. Moreover, HFNC has been applied in the setting of chronic respiratory failure (CRF) secondary to lung diseases, such as interstitial lung diseases (ILDs) and chronic obstructive pulmonary disease (COPD). There is also evidence of a significant beneficial effect of long-term domiciliary treatment with HFNC in patients with bronchiectasis [15,16]. HFNC has been used in mild hypercapnia secondary to COPD with promising results [17]. In particular, one metanalysis of three studies involving stable hypercapnic COPD patients demonstrated that long-term domiciliary treatment with HFNC reduced the exacerbation rate and improved the patients’ Quality of Life (QoL) compared with long-term COT, albeit without any significant change in the arterial oxygen (O_2_) and CO_2_ partial pressures (PaO_2_ and PaCO_2_, respectively) [18]. 

According to the 2022 European Respiratory Society practical guidelines [19], HFNC should be preferred over COT in ARF and over NIV in hypoxemic ARF; HFNC is equivalent to NIV in post-operative patients at high risk of pulmonary complications and is considered a second-line treatment, after trialing for NIV, in hypercapnic COPD patients.

Finally, recent evidence has shown an emerging role for HFNC in the setting of exercise training (ET) and pulmonary rehabilitation (PR) as a tool for improving endurance time and dyspnea scores in respiratory patients [20]. Therefore, the present narrative review aims to investigate the impact of HFNC on ET and PR with a thorough assessment of the current literature. 

## 2. Search Strategy

We performed a literature search in PubMed (https://pubmed.ncbi.nlm.nih.gov, accessed on 15 November 2023), Scopus (https://www.scopus.com, accessed on 15 November 2023), and Web of Science (https://www.webofscience.com, accessed on 16 November 2023) for articles regarding the application of HFNC during either a single exercise test performance or applied during multiple exercise sessions within an intensive PR program. The keywords used were the following: “High-Flow Nasal Cannula”, “HFNC”, “Pulmonary Rehabilitation”. Afterward, we screened the bibliographies of the selected articles for relevant citations. We included in the analysis original articles, metanalyses, and reviews of the scientific literature published between 1 January 2000 and 1 October 2023.

## 3. Rationale for the Application of HFNC during ET or PR

Benefits deriving from hyperoxia during the training of respiratory patients are currently still under discussion [21]. However, the rationale for O_2_ supplementation through HFNC during ET in respiratory diseases appears to be quite sound.

In COPD patients, factors such as static and dynamic hyperinflation, increased work of breath, and ventilatory demand might induce an earlier onset of dyspnea and physical exhaustion [22]. Patients with bronchiectasis often present COPD or asthma as comorbidities [23,24] and share with them several treatable traits, such as mucus plugs and airway obstruction. Both COPD and bronchiectasis eventually lead to CRF. The application of a warm and humidified airflow improves the airway clearance and the alveolar gas exchanges, with positive outcomes on QoL and a significant decrease in the exacerbation rates. In ILDs, alveolar gas diffusion is often impaired due to the thickening and fibrotic degeneration of the lung interstitium, which leads to systemic hypoxia, deconditioning, and earlier dynapenia [25]. In this clinical setting, HFNC has proven to be helpful as domiciliary treatment, with an improvement in lung mechanics [26] as well as an increase in the 6-minute walking distance (6MWD) and saturation recovery [27]. 

Despite the evident need for O_2_ supplementation in patients with CRF, COT is often poorly tolerated due to its action on the mucosal walls, altering the ciliary motion and drying out the airway secretions, sometimes to the extent of inducing painful ulcers. Moreover, COT does not seem to be as effective as HFNC in keeping the FiO_2_ constant [28].

**Figure 1:**
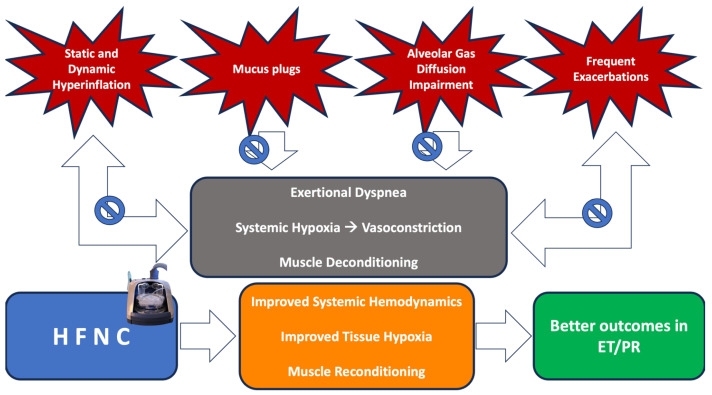
Several factors such as Static and Dynamic Hyperinflation, Mucus plugs, Alveolar Gas Diffusion Impairment, and Frequent Exacerbations contribute to the genesis of Exertional Dyspnea, Systemic Hypoxia, and Muscle Deconditioning. Thanks to its multiple mechanisms, HFNC therapy can affect such factors and thus induce an improvement in tissue oxygenation, systemic hemodynamics, and muscle reconditioning, thus leading to better outcomes in Exercise Training and Pulmonary Rehabilitation, in particular to an increase in the 6MWD and a reduction in the perception of dyspnea, while data on other outcomes are still uncertain. Abbreviations: 6MWD: 6-minute walking distance; ET, exercise training; HFNC, High-Flow Nasal Cannula; PR, pulmonary rehabilitation.

Although the evidence is still sparse, there are findings [29,30] which show that O_2_ supplementation during ET could be effective in contrasting the pathophysiological changes occurring during hypoxia in respiratory patients regardless of their basal condition. High-intensity interval training in a hyperoxic condition was demonstrated to improve mean peak work rate, and dyspnea and anxiety scores in COPD patients [31]. Such action could be due to a significant improvement in alveolar gas diffusion and a subsequent increase in the O_2_ availability in peripheral tissues, with beneficial effects on systemic hemodynamics and both voluntary and involuntary muscle work. While the former might be the direct consequence of the reduction of hypoxia-induced vasoconstriction, the latter might depend on a significant reduction of the metabolic distress otherwise induced by hypoxia. In brief, the more efficient the oxygen supplementation, the faster the muscle reconditioning and, consequently, the greater the improvement in exercise endurance (Figure 1). 

Considering the available evidence, it could be speculated that if HFNC is more reliable than COT in providing a constant FiO_2_, among all the other benefits, then exposing patients to HFNC during exercise might be linked to an improvement in the outcomes, as it has been already demonstrated with low-flow nasal cannula devices [31].

## 4. Effects of HFNC on ET and PR in COPD Patients

Several research groups investigated the effects of HFNC on both ET and PR in COPD patients.

Chao and colleagues, in a single-center, crossover trial [32] demonstrated that self-paced ET in COPD patients was improved by HFNC support. They enrolled 30 patients with a previous diagnosis of COPD and without any disability, who had undergone at least one month of PR. The patients were asked to perform a 6-minute walking test (6MWT) on two consecutive days after discharge. On the first day, each patient was randomly assigned either HFNC or COT support, while on the second day, they were assigned the opposite support. The mean difference in the 6MWD between the two repeated assessments was 27.3 m, a result which met statistical significance, although being slightly below the minimal clinically significant variation (at least 30 m, according to the American Thoracic Society/European Respiratory Society guidelines [33]). No significant variation was found in dyspnea assessed through the BORG scale, blood pressure (BP), respiratory rate (RR), and heart rate (HR) between the two groups. Nonetheless, the study highlights the positive response of COPD patients to HFNC during exercise and suggests that HFNC may have a role in improving their exercise performance.

The effects of HFNC on the rehabilitation outcome of chronic hypoxemic patients were evaluated in a study by Chihara et al. [34]. The authors enrolled 32 participants already in long term oxygen therapy (LTOT) at baseline, who underwent a four-week intensive PR training protocol. The patients were randomly assigned to the interventional group, which was characterized by the presence of HFNC at 50 L/min and FiO_2_ 100% during training, or to the control group, which received oxygen at the flow of 6 L/min through nasal cannula. All the patients underwent an incremental-load exercise test and three constant-load exercise tests (with daily LTOT, HFNC at 50 L/min FiO_2_ 100%, and COT at 6 L/min) before and after rehabilitation. The protocol was structured on exercises in five sessions per week on the same cycle ergometer with an initial setting at 80% of the maximum workload achieved at incremental-load testing. The study sample included 13 COPD patients (40.6%), of which six were assigned to the interventional group. After 4 weeks, the interventional group presented with a significant improvement in the 6MWT in terms of meters walked, with a mean difference of 55.2 ± 69.6 m versus −0.5 ± 87.3 m in the control group. In five out of six (83.3%) COPD patients assigned to the HFNC group, an increase of ≥30 m in the 6MWD was observed versus a proportion of one out of seven patients (14.3%) in the control group. Interestingly, in patients with severe hypoxemia at the baseline 6MWT (peripheral O_2_ saturation, SpO_2_ ≤ 85%), six out of six patients in the HFNC group improved ≥ 30 m in the 6MWT versus three out of seven (42.9%) in the control group, suggesting that HFNC might be considered as a first-choice treatment in the rehabilitation program of severe respiratory patients, regardless of their domiciliary oxygen support. In both groups, the duration of the constant-load exercise test increased at 4 weeks in comparison to baseline. In the HFNC group, however, this increase was maintained also when the patient underwent the test with the daily domiciliary LTOT prescription. This effect was not observed in the control group. No difference was found between the two groups in dyspnea assessed by BORG scale and maximum workload. However, the study presented with some limitations: the patients were elderly, with end-stage diseases, and quite heterogeneous. Therefore, the lack of improvement in constant-load exercise tests could be explained by the characteristics of the study sample.

Cirio et al. [35] evaluated the effects deriving from the use of HFNC during constant-load exercise testing in a crossover study. Twelve severe, stable, and ventilatory-limited COPD patients were enrolled. Ventilatory limitation was defined as a ratio between peak-minute ventilation and maximal voluntary ventilation <70% or 11 L/min. The patients underwent at first a symptom-limited incremental-load exercise test and, in the following days, two constant-load tests at 75% of the peak intensity exercise. One test was performed with the application of HFNC either at FiO_2_ 21% or with supplementation of oxygen in case of desaturation at the incremental-load exercise testing, while the other one was performed with a Venturi mask (VM) at the same FiO_2_. The HFNC group showed an increased exercise time, a better SpO_2_ throughout the entire effort, and, at isotime exercise, a lower perception of dyspnea and muscle fatigue, assessed through the BORG scale, compared with the control group in VM. Although it was only a pilot study with a small sample size, the authors conclude that HFNC can improve training endurance in COPD patients even without oxygen delivery.

A meta-analysis by Fu et al. [36] provided a summary of previous evidence concerning the role of HFNC on ET and PR in COPD patients. Of several hundred studies examined, only ten met the inclusion criteria, with a total of 600 patients analyzed. All the studies enrolled stable and elderly COPD patients who were treated with HFNC during ET. The primary outcomes were respiratory rate, forced expiratory volume in the first second (FEV_1_), tidal volume, PaO_2_, assessment of QoL by St. George Respiratory Questionnaire (SGRQ), 6MWT, and exercise endurance time. In two studies, the control group was on NIV, while the remaining were on COT. Among the data analyzed in six studies, there was evidence of a reduced RR in the group using HFNC during ET, but there was no difference in post-ET PaO_2_ between the HFNC and control groups. The SGRQ was reportedly improved in the HFNC groups in comparison with the COT control groups, while no differences were reported in comparison to the NIV subgroup. For the 6MWT, there was an increase in meters walked after the treatment with HFNC, however, without detecting an improvement in the overall exercise capacity.

Vitacca et al. [37] explored the role of HFNC during PR in patients with COPD and chronic hypoxia through a multicenter randomized-controlled trial. A total of 171 patients were enrolled in eight rehabilitation facilities and divided into two groups, one with O_2_ supplementation through HFNC, and the other with VM. Both groups underwent a 20-session ET with a cycle-ergometer at iso FiO_2_. The main outcomes were pre- and post-training endurance time and the 6MWD, while the secondary outcomes included QoL assessments. Although the HFNC group presented with a higher absolute value of endurance time at the end of the PR program, this difference was found to be not statistically significant. The only variable that reached statistical significance was the difference in the improvement at 6MWD. The authors, however, concluded that, albeit not statistically significant, most of the results of their study indicate that HFNC is a valid tool for the improvement of ET and its routine use in PR for COPD patients could lead to a clinically relevant improvement of their rehabilitation outcomes.

Volpi et al. [38] investigated the role of HFNC in COPD patients with nocturnal NIV who underwent an intensive program of PR. A total of 31 COPD patients were enrolled and completed a 60-session rehabilitation program; 15 patients were randomly assigned to the experimental group, which was characterized by the add-on of HFNC during ET. Patients were evaluated at baseline and after each 20 rehabilitation sessions. While the study did not meet significance for the primary outcome (∆6MWD between groups), some secondary outcomes seemed to be positively affected by HFNC: the BORG scale for dyspnea and fatigue, in fact, improved between the baseline and the last timepoint significantly more in the HFNC group. In light of their results, the authors conclude that, although HFNC cannot induce a statistically significant improvement in physical activity outcomes, it is able to improve both the subjective perception of dyspnea and fatigue and the dyspnea scores, with a considerable impact on QoL.

To summarize the aforementioned studies, HFNC seems to affect in a positive way some of the main outcomes related to physical exercise and PR. In fact, HFNC seems to be able to improve the performance of COPD patients in the 6MWT. This positive response to HFNC was observed in all the studies including the 6MWT among the outcomes, except one [38], in which the 6MWD did not significantly change between the interventional and the control group. However, the effect of HFNC on endurance time seems to be less clear, since it reached statistical significance only in one study [35], while in the remaining ones, it was substantially unchanged between HFNC and control groups. Dyspnea was found to be decreased in two studies [35,38] while in other two studies, it was found to be unaffected by HFNC [32,34]. Therefore, it can be stated that while the impact of HFNC on 6MWD and dyspnea is quite clear, its effects on endurance time are not yet defined. Finally, other outcomes, such as HR, RR, and BP seem to be unaffected by HFNC.

All the studies discussed above have been summarized in Table 1.

In conclusion, the use of HFNC has still several unclear aspects about its possible application to exercise training. Most of the uncertainty is related to the limitations of the available studies, which affect the final analysis for each proposed primary outcome; in fact, most studies present a high degree of heterogeneity in the samples and little or no stratification of the severity of COPD.

## 5. HFNC and PR in Other Respiratory Diseases

### 5.1. HFNC during ET and PR in ILDs

With fewer results in comparison with COPD, some research groups investigated the role of HFNC in the setting of ILDs.

Among the 32 patients evaluated by Chihara et al. [34], there were 15 subjects with interstitial pulmonary fibrosis (46.9%). As described above, patients were randomly assigned to the interventional group, which was characterized by the presence of HFNC at 50 L/min and FiO_2_ 100% during training, or to the control group, which received O_2_ at the flow of 6 L/min through nasal cannula. All patients underwent an incremental-load exercise test and three constant-load exercise tests (with daily LTOT, HFNC at 50 L/min FiO_2_ 100%, and OT at 6 L/min) before and after rehabilitation. The protocol was structured on exercises in five sessions per week on the same cycle ergometer with an initial setting at 80% of the maximum workload achieved at incremental-load testing. After 4 weeks, the interventional group presented overall with a significant improvement in the 6MWT in terms of meters walked, with a mean difference of 55.2 ± 69.6 m versus −0.5 ± 87.3 m in the control group. However, only in five out of nine (55.6%) pulmonary fibrosis patients assigned to the HFNC group an increase of ≥30 m in the 6MWD was observed, versus a proportion of four out of six patients (66.67%) in the control group. In both groups, the duration of the constant-load exercise test increased at 4 weeks in comparison to baseline.

Harada et al. [39] designed a randomized crossover trial that investigated the effect of applying HFNC in 24 patients during exercise testing in comparison to VM. All patients had been diagnosed with idiopathic pulmonary fibrosis (IPF) according to the 2018 ATS/ERS/JRS/ALAT guideline [40] and manifested exertional dyspnea and desaturation (SpO_2_ < 90% at the 6MWT) at baseline. All the patients enrolled underwent an incremental load exercise test and a baseline constant-load exercise test at 80% peak work. Afterward, they underwent the same test another time with HFNC and another with VM, in a random order, thus forming two groups (Arm A, with HFNC test done prior to VM, and Arm B, the opposite). The primary outcome was endurance time in the constant-load exercise test, while the secondary outcomes were SpO_2_, dyspnea, leg fatigue, heart rate, and comfort. The study met the primary outcome because the HFNC group had an increased endurance time than the VM one; the study also demonstrated that, when on HFNC support, patients showed a higher peripheral saturation and reduced leg fatigue. However, dyspnea, maximum heart rate, and comfort at 80% peak work rate were not affected by the application of HFNC.

Suzuki et al. [41] investigated how HFNC could alter the response to ET in patients diagnosed with fibrotic ILDs in comparison with VM. The study was a randomized crossover one and enrolled 20 patients who had only mild desaturation at the basal constant-work-rate endurance test (SpO_2_ ≥ 88%). All patients underwent a symptom-limited incremental exercise test; then, patients were randomly assigned to group A, where a high-intensity constant-work-rate endurance test was performed first with VM, and, on the following day, on HFNC; and group B, where the tests were performed in the reverse order. The primary outcome was endurance time, while the secondary outcomes were SpO_2_, HR, dyspnea assessed with the BORG scale, comfort, and adverse events. The study did not show any statistically significant differences between the two groups in all the outcomes. Neither the subgroup analysis of the “good responders” (defined as an improvement >100 s or 33% in endurance time in comparison to the baseline test) nor the stratification according to the presence of pulmonary hypertension demonstrated any superiority of HFNC on VM. These discouraging results, however, should be interpreted with caution, because the sample included patients with diverse kinds of ILDs with different underlying pathological mechanisms and systemic involvement.

To conclude, it can be stated that, in the setting of ILDs, the evidence supporting HFNC therapy for ET is sparse and inconsistent, given the limited number of studies and the high heterogeneity of the patients involved. However, one study [34] has demonstrated an improvement in the performance in the 6MWT, although not clinically significant, while dyspnea was substantially unaffected in all the three studies included in the current review [34,39,41]. This could be related to the fact that dyspnea in ILDs can be controlled by supplementing oxygen at higher FiO_2_ without a significant risk of hypercapnia, which, on the contrary, is an important limitation in COPD patients.

### 5.2. HFNC and ET in Patients with Primary or Secondary Lung Cancer

One study by Hui et al. [42] evaluated the role of the supplementation of O_2_ through HFNC or COT in 45 non-hypoxemic patients diagnosed with cancer and primary or secondary lung involvement. Patients were randomly assigned to one of the four investigational groups (HFNC + FiO_2_ 100%, HFNC without O_2_ enrichment, COT, and low flow air at 2 L/min). Patients in the HFNC + FiO_2_ 100% group improved dyspnea and leg discomfort at peak exercise as well as endurance time in constant-work-rate exercise testing. The study demonstrated a significant reduction in dyspnea in non-hypoxemic patients and an improvement in endurance time, but such intriguing results need to be confirmed by future larger trials.

The studies discussed above have been summarized in Table 2.

## 6. Limitations

The main limitation of the present review is related to the low number of original articles concerning our research question. Other limitations include the scarce homogeneity of the study populations and the limited amount of evidence concerning full cycles of intensive PR.

## 7. Conclusions

All the studies presented in the current review investigated the outcomes linked to the application of HFNC to either ET, mostly assessed through 6MWT and constant-workload exercise tests, or intensive PR programs. However, the evidence is still fragmentary and mostly based on single-center crossover studies, with study samples generally small and heterogeneous. This last aspect might explain the high degree of variability and non-significance of the results found by the different research teams.

However, there is evidence that the use of HFNC during exercise in respiratory patients can find some practical applications.

In COPD patients, the current evidence indicates that the application of HFNC during ET and PR programs is linked to an improvement in the performance in the 6MWT and has a positive impact on dyspnea; endurance time, however, seems to be only partially affected.

In ILD patients, HFNC has not shown a significant impact on dyspnea, apparently unaffected in all the three trials evaluated. Endurance time and the 6MWT, however, seemed to be at least partially improved by HFNC.

Lung cancer patients, evaluated in one single study, were overall good responders to HFNC, with a significant improvement in both endurance time and dyspnea in the group treated with HFNC and FiO_2_ 100%.

All these findings, albeit somewhat inconsistent, are, however, interesting if considered as starting points for novel trials, which should try to overcome the limitations represented by the small study samples and the excessive degree of heterogeneity within experimental groups. This could be achieved through multicenter trials and a meticulous stratification of the patients according to the severity of their disease.

Moreover, no study has so far focused on the role of HFNC in intensive PR of patients with ILDs, bronchiectasis, and lung cancer, although the sparse evidence regarding ET appears to be promising.

In conclusion, as of today, HFNC seems to be linked to an improvement in physical performance, measured as 6MWD, as well as subjective perception of dyspnea and fatigue in constant-load exercise tests. However, the extent of such improvement is still debatable, and new studies are needed to fill the current research gap.

## Figures and Tables

**Table 1:** Studies assessing the role of HFNC in the ET/PR of COPD patients.

Study	Subjects	Design	Intervention	Performance Tests	Main Findings
Chao et al. (2021) [32]	30 stable COPD patients at discharge from PR	Randomized crossover trial	HFNC vs. COT	6MWT	↑ 6MWD *, ↔ Dyspnea, ↔ HR, ↔ BP, ↔ RR
Chihara et al. (2022) [34]	13 COPD patients with CRF	Single-center RCT/crossover trial	HFNC at 50 L/min and FiO_2_ 100% vs. Oxygen at 6 L/min through nasal cannula during PR	6MWT, Constant-load exercise testing at 80% maximal capacity	↑ 6MWD, ↔ Endurance time, ↔ Dyspnea, ↔ HR, ↔ BP, ↔ RR
Cirio et al. (2016) [35]	12 severe, stable, ventilatory-limited COPD patients	Randomized crossover trial	HFNC vs. VM at the same FiO_2_	Constant-load exercise testing at 75% maximal capacity	↑ Endurance Time, ↑ SpO_2_, ↓ Dyspnea
Fu et al. (2020) [36]	600 Stable COPD patients from eight studies	Metanalysis	HFNC vs. COT (six studies) HFNC vs. NIV (two studies)	6MWT, Constant-load exercise testing	↓ RR ^, ↔ PaO_2_, ↑ SGRQ, ↑ 6MWD, ↔ Endurance Time
Vitacca et al. (2020) [37]	171 COPD patients undergoing PR	Multicenter RCT	HFNC vs. VM during PR	6MWT, Constant-load exercise testing	↑ 6MWD, ↔ Endurance Time
Volpi et al. (2022) [38]	31 COPD patients with nocturnal NIV undergoing PR	Single-center RCT	HFNC vs. COT during PR	6MWT, Constant-load exercise testing	↔ 6MWD, ↓ Dyspnea, ↓ Fatigue

* Statistically, but not clinically significant. ^ Only vs. COT; the variation vs. NIV was not significant.↑ = significantly increased vs. control group (*p* < 0.05); ↔ = no significant difference vs. control group (*p* > 0.05); ↓ = significantly reduced vs. control group (*p* < 0.05). Abbreviations: 6MWD, 6-minute walking distance; 6MWT, 6-minute walking test; BP, blood pressure; COPD, chronic obstructive pulmonary disease; COT, conventional oxygen therapy; CRF, chronic respiratory failure; FiO_2_, fraction of inhaled oxygen; HFNC, high-flow nasal cannula; HR, heart rate; NIV, non-invasive ventilation; PaO_2_, arterial partial pressure of oxygen; PR, pulmonary rehabilitation; RCT, randomized controlled clinical trial; RR, respiratory rate; SRGQ, Saint George’s Respiratory Questionnaire; VM, venturi mask.

**Table 2:** Studies assessing HFNC in the clinical setting of ILDs and lung cancer.

Study	Subjects	Design	Intervention	Performance Tests	Main Findings
Chihara et al. (2022) [34]	15 IPF patients with CRF	Single-center RCT/crossover trial	HFNC at 50 L/min and FiO_2_ 100% vs. Oxygen at 6 L/min through nasal cannula during PR	6MWT, Constant-load exercise testing at 80% maximal capacity	↑ 6MWD, ↔ Endurance time, ↔ Dyspnea, ↔ HR, ↔ BP, ↔ RR
Harada et al. (2022) [39]	24 IPF patients with exertional dyspnea and desaturation	Randomized crossover trial	HFNC vs. VM at the same FiO_2_	Constant-load exercise testing at 80% maximal capacity	↑ Endurance Time, ↑ SpO_2_, ↓ Leg Fatigue, ↔ Dyspnea, ↔ HR, ↔ Comfort
Suzuki et al. (2020) [41]	20 fibrotic ILDs patients with mild exertional desaturation	Randomized crossover trial	HFNC vs. VM at the same FiO_2_	Constant-load exercise testing	↔ Endurance Time, ↔ SpO_2_, ↔ Dyspnea, ↔ HR, ↔ Comfort
Hui et al. (2021) [42]	45 non-hypoxemic patients with primary or secondary Lung Cancer	Single-center RCT	HFNC with FiO_2_ 100% vs. HFNC without O_2_ vs. COT vs. Low-Flow Air at 2 L/min	Constant-load exercise testing	↑ Endurance Time ^+^, ↓ Dyspnea ^+^

^+^ HFNC + FiO_2_ 100% vs. other groups. ↑ = significantly increased vs. control group (*p* < 0.05); ↔ = no significant difference vs. control group (*p* > 0.05); ↓ = significantly reduced vs. control group (*p* < 0.05). Abbreviations: 6MWD, 6-minute walking distance; 6MWT, 6-minute walking test; BP, blood pressure; COT, conventional oxygen therapy; CRF, chronic respiratory failure; HFNC, high-flow nasal cannula; HR, heart rate; ILDs, interstitial lung diseases; IPF, idiopathic pulmonary fibrosis; PR, pulmonary rehabilitation; SpO_2_: peripheral oxygen saturation; RCT, randomized controlled clinical trial; RR, respiratory rate; VM, venturi mask.

## Data Availability

Not applicable.
